# On the Indiscriminate Use of Alcoholic Stimulants in Disease

**Published:** 1867-09

**Authors:** Samuel Wilks

**Affiliations:** Physician to, and Lecturer on Medicine, at Guy’s Hospital


### Miscellaneous.

### On the Indiscriminate Use of Alcoholic Stimulants in Disease.

BY SAMUEL WILKS, M. D.,

Physician to, and Lecturer on Medicine, at Guy's Hospital.

In a clinical lecture delivered at Guy's Hospital on the above subject, Dr. Wilks said, "To my mind, the most important question in therapeutics at the present day is the value of alcohol in disease. If it be said that its frequent use is an evidence of its potency, this is the more sufficient reason why its administration sbould be watched with the extremest care. Like other drugs, it may be beneficial, useless, or harmful. Fevers will do well without this remedy. So wedded, however, are some to the idea of the absolute necessity of stimulants, that they have expressed almost incredulity when they have heard it stated that fevers will terminate favorably without them. Of course stimulants are often needed; but young persons with typhus and typhoid do far better, I believe, without them. That they make good recoveries on simple milk diet is a fact which my hospital cases prove, and which no arguments can gainsay; and, on the other hand, I have seen a marked improvement take place in some cases where a stimulus has been left off. It is also a fact that in bronchitis I have repeatedly seen improvement after stimulants have been omitted; and, as regards heart-disease, I am convinced that the amount of mischief done by stimulants is immense. In the case of fevers and bronchitis, the weak pulse is often but an indication of extreme capillary congestion, and a stimulus to the heart only aggravates the evil; and in the case of a diseased and weak heart, where repose is indicated, a constant stimulation by alcohol adds immensely to its trouble.

"It causes me daily surprise to observe how the effects of stimulation are overlooked. Often have I been called to see a patient apparently dying, sometimes of a nervous disorder, at another time of a liver complaint, and at another of heart-disease. He is lying in bed, where he (or she) has been for some time, and kept alive (as it is said) by brandy; the breath is abominable fetid; the heart's action is so rapid that it is impossible to say whether the organ is diseased or not; the patient refuses food, or if this be taken, it is rejected, and so he is plied with brandy to keep him alive; the body is, in fact, saturated with spirit, or its elements. My first remark on seeing such a case is, that a man cannot live on alcohol; he must take some food, or he will die. The correctness of such common-sense remarks is admitted, but qualified with the statement that no solids can be taken, and that if stimulants be omitted, it is feared the patient will sink. It is assumed that the constant administration of brandy is necessary for the temporary maintenance of life, and the idea never seems to have been conceived that the stimulation of the heart causes the weak, fluttering pulse, and the stimulation of the stomach a subacute gastritis. Do you ask me what method I adopt? The simplest possible. I withdraw every drop of the stimulant, and in a few hours the irritated stomach is partly restored to its normal condition, the nervous excitement abates, the patient takes a little food, and begins to mend. Do you ask again, whether I do not fear any frightful results from the sudden withdrawal of the stimulus? I say, not the least; I have no fear of the consequences. Not of delirium tremens? Not in the least. This is a disease not induced by the withdrawal of stimulants, but, on the contrary, is produced by a recent debauch. For the production of delirium tremens the patient must have been such an habitual tippler as to have weakened his brain, and must then have had an overdose of the stimulant to set up the disease. There are no facts to show that the withdrawal of the accustomed drink is attended with any evil results, although I know that an imaginary fear of this kind leads to an erroneous and vicious method of treatment—the plying the patient with a stimulant during the violence of the attack, the effect of which is to prevent or prolong the cure. Rest and repose, with the avoidance of stimulation, is the treatment which the patient requires. The success of digitals may be mentioned in corroboration of this view. I repeat that there are no facts to show that delirium tremens is produced by the withdrawal of stimulants, whilst it is a fact, as I could illustrate by many cases, that nothing but good results from its absolute discontinuance in the desperate cases to which I have alluded.

"That many cases of disease of various kinds would do far better without stimulants I am perfectly confident. But lately I have seen the case of a gentleman, about sixty years of age, who passed through a most severe attack of pneumonia without the use of stimulants. He had been a tolerably free liver, and would not have been called a good subject; but having before me the case of another gentleman of the same age, who had just died of pneumonia, and who had taken a large lot of brandy, I readily acquiesced in the patient's own view, that none should be given. It is very remarkable what extremes we have reached, and on how slight a scientific basis is founded the treatment of pneumonia. Not many years ago the antiphlogistic method was adopted, including bleeding, antimony, calomel, etc.; then came the 'let alone' method, and now we have the brandy treatment. What the need of this can be with Professor Hughes Bennett's statistics before us, I do not comprehend. My own opinion is (but of course this is only an opinion,) that in any given number of cases a larger majority would recover under the old antiphlogistic treatment than by the more modern method by brandy. As regards heart-disease, the utmost discrimination is required in the use of stimulants. There are cases where an undoubted benefit is produced by them; but there are others, and these I have seen repeatedly, where alcohol has induced palpitation, fluttering, great distress, and constant sleepless nights, but where, on the other hand, the withdrawal of the spirit, and the substitution of a dose of digitalis or henbane, has been of the most essential service. The administration of a stimulus, in the attempt to overcome disease, in lieu of good and well-tried remedies, evinces the very worst form of medical skepticism with which I am acquainted.

"It is not only in these severe cases of disease, but in lesser troubles, that your recommendation of stimulants may do incalculable mischief. You visit, for example, an ailing lady, and she details to you a number of troubles of a nervous and dyspeptic character. She is sitting in-doors all day, taking no exercise, living well, and consequently drifting into a weak and flabby condition. You place your hand on her pulse, and, finding it feeble, condole with her on her state of health, assure her that she does not live well enough, and order her a few extra glasses of wine or a little brandy.* You find that she grows no better for the advice; but perhaps you never reflect that you have been adding fuel to the fire. Knowing not what to do in the way of treatment, you order her out of town, and she immediately begins to improve. She goes to Brighton, rides on horseback, or walks miles a day on the Parade, regains her appetite, craves less for stimulants, and her health is restored. If, on the contrary, you fail to remove her from her home, she goes on from bad to worse; she takes to her bed, eats less food, drinks more wine and brandy, until, having become one mass of fatty degeneration, life can hold no longer, and death ends the scene. This lady has been killed with kindness. This is no imaginary case; my mind's eye is carrying me to the bedside of more than one such instance. Do not then assume that alcohol is an equivalent to a tonic, and that it must be necessarily administered because your patient is weak. It may be that that very weakness is due to the long-continued pernicious effects of this same stimulant; indeed, as you have often heard me say in the out-patient room, if a man comes into our presence with a tottering gait, bloated face, and his nervous energy all gone, you may be quite sure that he has been taking 'strengthening' things all his life "—*Lancet*, April 27, 1867.

* The word "little," it must be remembered, has long ceased to maintain its original signification in reference to eating, drinking, and physicking. It would be extremely vulgar were we to be asked at our dinner table to take otherwise than a "little" more; and the doctor would not be forgiven by his patient were he, in detailing the ingredients of his prescription, to state that he had administered the regular dose, but that he had given only a "little" of this or that. When therefore a patient is ordered a "little" brandy, the objection in no way qualifies the amount.

